# Optimal Complementary Feeding Practices and Determinants Among 6–23 Months Aged Children’s Mothers in Kedida Gamela District and Durame Town, Southern Ethiopia: Cross‐Sectional Comparative Study

**DOI:** 10.1155/jnme/5360358

**Published:** 2026-05-27

**Authors:** Yididia Desta Langena, Diliab Desta Langena, Minale Fekadie Baye, Alemayehu Atomsa Sona, Melese Sinaga Teshome

**Affiliations:** ^1^ Applied Human Nutrition Department, Hawassa University, Hawassa, Ethiopia, hu.edu.et; ^2^ Biomedical Sciences Department, Jimma University, Jimma, Ethiopia, ju.edu.et; ^3^ Biomedical Science Department, Jimma University, Jimma, Ethiopia, ju.edu.et; ^4^ Epidemiology and Biostatistics Department, Jimma University, Jimma, Ethiopia, ju.edu.et; ^5^ Nutrition and Dietetics Department, Jimma University, Jimma, Ethiopia, ju.edu.et

**Keywords:** child health, complementary feeding, nutrition, optimal feeding practices, Southern Ethiopia

## Abstract

**Background:**

For children to grow and develop to their full potential, the first 2 years of life are critical. Infectious disease incidence and associated mortality can be reduced by optimal complementary feeding practices. Studies on these practices and determinants, however, are few in this field. The purpose of this study is to close this information gap.

**Methods:**

Between January 15 and February 15, 2021, 565 mothers of children ages 6 to 23 months participated in a community‐based comparative cross‐sectional study in Durame town and Kedida Gamela district. Structured questionnaires were used to gather data through in‐person interviews, and SPSS Version 25 was used for analysis. Determinants of complementary feeding practices were identified through bivariate and multivariable logistic regression, utilizing adjusted odds ratios with a 95% confidence interval *p* value 0.05.

**Results:**

Complementary feeding practice magnitudes in rural and urban areas were 13.1% and 25.1%, respectively. The mother’s knowledge of good complementary feeding (AOR = 5.13, 95% CI: 1.68, 15.65) and household food insecurity (AOR = 3.5, 95% CI: 1.4, 8.75) was associated with optimal complementary feeding practices in rural areas, while child age (AOR = 3.57, 95% CI: 1.08, 11.805), postnatal care (AOR = 2.58, 95% CI: 1.28, 5.16), and the mother’s awareness about complementary feeding practices (AOR = 4.5, 95% CI: 1.13, 14.17) were associated with good complementary feeding practices in urban settings.

**Conclusion and Recommendation:**

In comparison with rural areas, the magnitude of optimal complementary feeding practice is significantly higher in urban areas. Integration of nutrition education and infant and young child feeding into maternal and child health services is essential to improving child feeding practices.

## 1. Introduction

Children’s development depends on adequate nutritional intake. During the first 2 years of life, it is critical to maintain optimal growth and development [1, 2]. Babies grow faster than ever during this period, and they require adequate nutrition [2, 3].

Breast milk is insufficient to meet the energy and nutritional needs of developing infants and children after 6 months of age. The World Health Organization (WHO, 2013) stated that early breastfeeding initiation and timely introduction of complementary foods are essential for optimal child development and growth. In order to satisfy the nutritional needs of six‐month‐old infants, these foods should be provided regularly, in sufficient quantities, and with a range of options. For healthy growth, appropriate organ formation and function, a robust immune system, and neurological and cognitive development, breastfeeding must continue for up to 2 years [4, 5]. Effective complementary feeding supports children’s mental and motor development and helps prevent obesity and metabolic issues later in life [2].

Both human development and economic growth depend on a healthy population capable of acquiring essential skills, engaging in critical thinking, and making meaningful contributions to the development of their communities. Malnourished children are less able to lead productive lives and have cognitive deficits, both of which contribute to poverty [6, 7].

Children under two suffer negative health effects from inadequate complementary diets, poor feeding practices, and high infection rates. Even with optimal breastfeeding, a lack of dietary variety and frequent meals after 6 months can lead to stunting. The low quality and variety of foods significantly impact their growth and nutritional status [8].

Each year, three million children around the world die from undernutrition, which contributes to 45% of all deaths among children. Inappropriate feeding practices contribute to over two‐thirds of infant deaths occurring within the first year of life [9].

In sub‐Saharan Africa, including Ethiopia, malnutrition affects millions of children and has a significant burden. The prevalence accounts for stunting 8.8% to 64.5, wasting from 1.4% to 55.8%, and underweight accounts 16.4% [10].

Dietary diversity is a critical factor impacting malnutrition rates, with children experiencing inadequate minimum dietary diversity at greater risk of being wasted, overweight, or stunted [11]. A child’s chances of survival are decreased by stunting and other forms of malnutrition, which also impede optimal growth and health [12]. Besides, it adversely affects brain development and leads to long‐term consequences on future earnings, academic performance, and cognitive abilities. These ultimately hinder national development [13].

Less than one‐third of children aged 6–23 months who live in Africa fulfill basic nutritional diversity requirements, and fifty percent do not meet meal frequency standards. This inadequacy increases the risk of stunted growth, especially in the first 2 years of life by threefold [9].

A recent analysis of data from 21 countries highlights that poor dietary practices regarding complementary foods are linked to negative growth trends for children within many developing nations [14]. In sub‐Saharan Africa, the rates of infant and child mortality are exacerbated by inadequate feeding practices, poor quality complementary foods, micronutrient deficiencies, and frequent infections, highlighting a critical public health concern [15].

In Ethiopia, child health has been prioritized under the Ethiopian Health Care Development Program [16]. However, the prevalence of appropriate complementary feeding practices among children aged 6–23 months remains critically low, falling below international recommendations [17]. Evidence from the Ethiopia Demographic and Health Survey 2016 indicates that only 7% of children in this age group receive diets that meet acceptable standards, with a notable disparity between urban (19%) and rural (6%) populations. While 60% of children received food, only 45% were fed the required number of times, and just 14% had a variety of food. This indicates that, despite some complementary feeding, many children are still receiving low‐quality, undiversified food [18].

The government of Ethiopia has established a goal to eliminate undernutrition in children by 2030 through the “Seqota” Declaration. It is committed to investing in nutrition for health and sustainable development, involving multiple sectors to prioritize six key behavior changes [19]. The Ethiopian National Nutrition Program is designed to monitor child feeding practices and implement evidence‐based interventions to improve adherence to recommended practices [17, 20].

To advance optimal complementary feeding practices in this crucial time period of child development and growth, it is essential to measure these practices and identify their associated factors. In Durame town and the Kedida Gamela district, the extent of complementary feeding practices is not well documented, especially in a comparative manner between urban and rural areas.

Thus, the study was conducted to assess the magnitude of optimal complementary feeding practices and its key influencing factors among children aged 6–23 months using core indicators and cross‐sectional comparative study design.

## 2. Methods

### 2.1. Study Setting

This research was undertaken in Durame town and Kedida Gamela Woreda in the Kembata Tembaro province, Ethiopia. Durame, located 306 km south of Addis Ababa, has a population of 51,453, comprising 23,456 women and 26,876 men, including 1802 children aged 6–23 months. The district experiences yearly temperatures between 15°C and 20°C and receives 1001–1400 mm of rainfall, with agriculture as the primary livelihood source. The major crops include wheat, barley, fruits, and vegetables. Healthcare facilities in Durame comprise one district hospital, three health centers, one health post, and nine private clinics. Kedida Gamela, a rural district near Durame, has a population of 70,978 (36,199 men and 34,779 women), with two health centers, twelve health posts, and three private clinics. Key crops within the district also include wheat, barley, teff, and maize, while kocho and kita are commonly consumed foods.

### 2.2. Study Design and Study Period

A cross‐sectional comparative study was undertaken in a community setting between January 15 and February 15, 2021.

### 2.3. Population of Interest

#### 2.3.1. Source Population

All mothers and/or caretakers having a child aged 6–23 months and who were living in Durame town, Kedida Gamela district for at least 6 months.

#### 2.3.2. Study Population

Mothers or caretakers having a child aged 6–23 months who were included in this study from selected Kebeles of urban and rural areas.

### 2.4. Eligibility Criteria

Mothers or caretakers having a child 6–23 months of age who lived in rural and urban parts of the study are included for at least six months.

### 2.5. Exclusion Criteria

A mother or caretaker who was critically ill, unable to communicate, and/or had an ill child during the data collection period was excluded.

### 2.6. Sample Size Determination

The sample size for the first research objective was calculated using a double‐population proportion formula. The assumptions included a 95% confidence level, 80% power, and proportion of optimal complementary feeding practice (11.4%) from a study conducted in the rural area of Damot Sore district [21] and 26.8% from a study in urban Ambo town [22]. A design effect of 1.5 and a 10% nonresponse were also considered. Based on these parameters, the final sample size was determined to be 565 participants, of which 279 were urban and 277 were rural residents.

### 2.7. Participant Selection Procedure

Multistage sampling approach was employed for choosing study subjects. The selected two districts were Durame Town and Kedida Gamela District, taken purposefully from the Kembata Tembaro Zone. The stratification was based on key characteristics, categorizing the districts as urban or rural. Durame Town comprises three urban kebeles, while Kedida Gamela District contains eleven rural kebeles.

From the urban area, two kebeles were chosen, and four kebeles were selected from the rural area employing a simple random sampling approach. The overall sample size was then allocated proportionally across the sampled kebeles. Participants within each kebele were identified using a systematic sampling approach, beginning with a randomly selected first household and subsequently selecting every k^th^ household based on a predetermined sampling interval. One eligible child and their mother who was present at the time of the survey were chosen from each household. A lottery method was used to select one child at random from households with two children between the ages of six and twenty‐three months.

### 2.8. Data Collection Process, Quality Control, and Instruments

A systematic questionnaire was used to collect data through in‐person interviews. The questionnaire was initially written in English, translated into Amharic and Kamabatissa affoo, and then back‐translated to ensure consistency.

The questionnaire was adapted from WHO guidelines and previous studies on complementary feeding practices. Two health professional supervisors and six data collectors were trained for 2 days, with daily checks for completeness and on‐the‐spot corrections.

Children’s food intake and dietary diversity were assessed using a 24‐h dietary recall method. Mothers/caregivers were asked to report all food groups consumed the day before. Seven WHO food groups were used to compute the dietary diversity score. These are grains, roots, and tubers; legumes and nuts; dairy products; flesh foods; eggs; vitamin A‐rich fruits and vegetables; and other fruits and vegetables. Each food group received a score of “1” for consumption and “0” for nonconsumption.

A Household Food Insecurity Access Scale (HFIAS) was used to categorize household food insecurity as food secure, mildly food insecure, moderately food insecure, and severely food insecure [23]. However, for this study, households were considered either food secure, if classified as such by HFIAS, or if classified as mildly, moderately, or severely food insecure.

### 2.9. Data Processing and Analysis

SPSS Version 25 software was employed for data entry. To describe the sociodemographic and other features of the respondents, descriptive statistics, frequency distribution, and percentage were computed and presented in tables. To assess whether there were any notable differences in the variables between children in rural and urban areas, the chi‐square test was used. To determine the relationship between each independent variable and the dependent variable, bivariate logistic regression analysis was used. Variables that showed a *p* value of less than 0.25 in the bivariate analysis were entered into the multivariable analysis. A multivariable binary logistic regression model was used to control for cofounders that have an association with both exposure and outcome variables and independently produce an effect on the outcome. The model has controlled the effect of both covariates and confounders by including all confounders in the model simultaneously, and it fixes their effect. The output of the model was interpreted using adjusted odds ratio (AOR), as the value of AOR has been adjusted for confounders by the model. Finally, statistical association was determined at *p* value less than 0.05 and an AOR with 95% confidence interval.

### 2.10. Operational Definition

Complementary feeding is the process of introducing appropriate solid and/or semisolid foods and liquids to an infant whose nutritional needs cannot be satisfied by breast milk alone, starting at 6 months of age. Breastfeeding is continued until the child is 2 years of age or older [24].

Optimal complementary feeding is providing breast milk or suitable substitutes along with solid, semisolid, or soft foods to children aged 6–23 months, following recommended feeding indicators. A child achieves adequate complementary feeding by meeting three criteria: (1) minimum dietary diversity, involving foods from recommended groups (grains, legumes, dairy, flesh foods, eggs, vitamin A–rich fruits, and vegetables); (2) minimum meal frequency, requiring at least 2 meals daily for breastfed children aged 6–8 months, 3 for those aged 9–23 months, and 4 for non‐breastfed children; and (3) minimum acceptable diet, which includes timely introduction of complementary foods at 6 months, continued breastfeeding or suitable alternatives, and meeting the diversity and frequency requirements [25]. A binary response (yes or no) was used to measure optimal complementary feeding. When a mother or caregiver meets all three requirements of optimal feeding, the answer is “yes”; otherwise, it is “no.”

Timely initiation of solid, semisolid, or soft foods was defined as the introduction of complementary foods to infants at 6 to 23 months of age, in accordance with the WHO recommendation. It was measured as timely (at 6 months), early (before 6 months), or late (after 6 months) based on maternal or caregiver reports of when complementary foods were first introduced.

Minimum diet diversity was defined as the proportion of children aged 6–23 months who received foods and beverages from at least five out of eight defined food groups during the 24 h preceding the survey. Dietary diversity was categorized as adequate (≥ 5 food groups) and inadequate (< 5 food groups).

Minimum meal frequency refers to the percentage of breastfed and non‐breastfed children aged 6–23 months who received solid, semisolid, or soft foods four times in the past 24 h preceding the survey and measured as “Adequate” if they meet the recommended meal frequency (≥ 4 times for non‐breastfed children and an age‐appropriate frequency for breastfed children) and “Inadequate” if they do not.

Minimum acceptable diet: Minimum acceptable diet for children aged 6–23 months was defined as receiving at least two milk feedings and adherence to both the minimum dietary diversity and minimum meal frequency criteria within the previous 24 h. Based on mothers’ or caregivers’ responses, children were classified as either meeting or not meeting the minimum acceptable diet. Children who met both minimum dietary diversity and minimum meal frequency were considered to meet the MAD, whereas those who failed to meet either criterion were classified as not meeting the MAD.

Sufficient knowledge is the mother’s understanding level, determined by a knowledge assessment score. This assessment is conducted using a structured questionnaire where responses were scored to derive a total knowledge score for each mother. Mothers whose total knowledge scores are equal to or above the mean score of the study population were classified as having sufficient knowledge of optimal complementary feeding practices, whereas those scoring below the mean were considered to have insufficient knowledge.

Insufficient knowledge is a mother’s limited level of understanding of complementary feeding practices and was measured by a structured knowledge assessment questionnaire. Individual responses were scored and aggregated to generate an overall knowledge score for each respondent. Mothers whose total knowledge scores fell below the mean score of the study population were classified as having insufficient knowledge of optimal complementary feeding practices.

## 3. Study Findings

### 3.1. Study Participants’ Sociodemographic and Socioeconomic Characteristics

Five hundred and fifty‐six mothers with children within 6–23 months of age enrolled in the study, yielding an overall response rate of 98.8% (98.2% rural, 98.8% urban). The respondents’ mean age was 28.75 years (SD ± 4.6) among rural participants and 27.5 years (SD ± 3.9) among urban participants. Most respondents were married (96.8% rural, 97.1% urban) and predominantly protestant (86.4% rural, 84.1% urban). Educationally, 35.1% of rural and 20.9% of urban mothers had a primary school education, while 30.5% of rural households and 67.9% of urban households reported food security. The majority were housewives (82.8% rural, 43.0% urban), and over half of the respondents had family sizes ranging from 4–6 (54.8% rural, 65.7% urban) (Table 1).

**TABLE 1:** Children’s mothers’ sociodemographic and socioeconomic characteristics.

Variables	Rural *n* (%)	Urban *n* (%)	*X* ^2^	*p* value
Mothers age				
< 20	6 (2.2%)	9 (3.2%)	63.704	< 0.001
20–24	50 (17.9%)	68 (24.5%)		
25–29	67 (24.0%)	126 (45.5%)		
30–34	104 (37.3%)	67 (24.2%)		
≥ 35	52 (18.6%)	7 (2.5%)		
Mother’s marital status				
Married	270 (96.8%)	269 (97.1%)	0.752	0.687
Divorced	5 (1.8%)	6 (2.2%)		
Widowed	4 (1.4%)	2 (0.7%)		
Mother’s religion				
Protestant	241 (86.4%)	233 (84.1%)	1.232	0.745
Orthodox	20 (7.2%)	22 (7.9%)		
Catholic	16 (5.7%)	21 (7.6%)		
Muslim	2 (0.7%)	1 (0.4%)		
Mother’s educational level				
Unable to read and write	72 (25.8%)	19 (6.9%)	140.641	< 0.001
Read and write	69 (24.7%)	34 (12.3%)		
Primary school	98 (35.1%)	58 (20.9%)		
Secondary school	36 (12.9%)	91 (32.9%)		
College/university	4 (1.4%)	75 (27.1%)		
Mother’s occupational category				
Housewife	231 (82.8%)	119 (43.0%)	122.961	< 0.001
Daily laborer	19 (6.8%)	18 (6.5%)		
Merchant	27 (9.7%)	59 (21.3%)		
Government worker	2 (0.7%)	81 (29.2%)		
Father’s educational level				
Unable to read and write	44 (16.3%)	4 (1.5%)	215.429	< 0.001
Read and write	49 (18.1%)	21 (7.8%)		
Primary school	85 (31.5%)	25 (9.3%)		
Secondary school	85 (31.5%)	64 (23.8%)		
College/university	7 (2.9%)	155 (57.6%)		
Father’s occupational category				
Governmental worker	9 (3.3%)	151 (56.1%)	334.9	< 0.001
Farmer	182 (67.4%)	0		
Merchant	22 (8.1%)	71 (26.4%)		
Day labor	48 (17.8%)	41 (15.2%)		
Other	9 (3.3%)	6 (2.2%)		
Head of household				
Husband	270 (96.8%)	268 (97.1%)	0.050	0.823
Wife	9 (3.2%)	8 (2.9%)		
Family size				
1–3	20 (7.2%)	57 (20.6%)	53.394	< 0.001
4–6	153 (54.8%)	182 (65.7%)		
≥ 7	106 (38.0%)	38 (13.7%)		
Monthly income				
< 999	113 (40.5%)	3 (1.1%)	377.438	< 0.001
1000–1999	123 (44.1%)	21 (7.6%)		
2000–2999	37 (13.3%)	37 (13.4%)		
3000–3999	6 (2.2%)	44 (15.9%)		
≥ 4000	0 (0.0%)	172 (62.1%)		
Household food security status				
Food secure	85 (30.5%)	188 (67.9%)	77.812	< 0.001
Food insecure	194 (69.5%)	89 (32.1%)		
Source of drinking water				
Piped water	12 (4.3%)	264 (90.6%)	506.467	< 0.001
Public tab	249 (89.2%)	23 (8.3%)		
Protected well	18 (6.5%)	3 (1.1%)		
Type of toilet facility				
Traditional latrine	242 (86.7%)	37 (13.4%)	374.412	< 0.001
Latrine with shade	28 (10.0%)	240 (86.6%)		
No toilet	9 (3.2%)	0 (0.0%)		
Solid waste disposal				
On‐field	100 (35.8%)	0 (0.0%)	270.615	< 0.001
Solid waste disposal	102 (36.6%)	73 (26.4%)		
Garbage container	0 (0.0%)	158 (57.0%)		
Burn	77 (27.6%)	46 (16.6%)		

### 3.2. Maternal Obstetric and Child‐Related Characteristics

In rural areas, 48.4% of children were female, compared to 52.3% in urban areas. Approximately 66.3% of rural children and 55.6% of urban children were aged 12–23 months, with mean ages of 15.3 ± 5.4 months and 14.2 ± 5.3 months, respectively. Most participants (93.9% in rural and 98.9% in urban areas) attended antenatal care at least once during their last pregnancy, but fewer than half (44.9%) in urban areas had four visits. Nearly all respondents (97.4% in rural and 99.9% in urban areas) delivered at health facilities. Postnatal care was accessed by 23.7% of rural and 62.5% of urban participants, with 86.4% of those in rural and 90.2% in urban areas receiving information on complementary feeding practices (Table 2).

**TABLE 2:** Children’s mothers’ maternal, obstetric, and child‐related characteristics.

Variables	Rural *n* (%)	Urban *n* (%)	*X* ^2^	*p* value
Child sex				
Male	135 (48.4%)	145 (52.3%)	0.872	0.350
Female	144 (51.6%)	132 (47.7%)		
Child age				
6–8 months	48 (17.2%)	32 (11.6%)	20.809	< 0.001
9–11 months	46 (16.5%)	91 (32.9%)		
12–23 months	185 (66.3%)	154 (55.6%)		
ANC follow‐up				
Yes	262 (93.9%)	274 (98.9%)	10.062	0.002
No	17 (6.1%)	3 (1.1%)		
How many times has the patient visited ANC followed up				
One times	18 (6.9%)	6 (2.2%)	83.178	< 0.001
Two times	109 (41.6%)	58 (21.2%)		
Three times	107 (40.8%)	87 (31.8%)		
Four times	28 (10.7%)	123 (44.9%)		
Place of delivery				
Health center	152 (54.5%)	88 (31.8%)	36.542	< 0.001
Hospital	117 (41.9%)	186 (67.1%)		
Home	10 (3.6%)	3 (1.1%)		
Mode of delivery				
Spontaneous delivery	265 (95.0%)	186 (67.1%)	70.299	< 0.001
Cesarean section	14 (5.0%)	91 (32.9%)		
Postnatal care				
Yes	66 (23.7%)	173 (62.5%)	85.377	< 0.001
No	213 (76.3%)	104 (37.5%)		
During PNC, information about CFP				
Yes	57 (86.4%)	156 (90.2%)	4.160	0.041
No	9 (13.6%)	17 (9.8%)		
Eat additional meals every day during pregnancy and lactation				
Yes	75 (26.9%)	223 (81.4%)	165.272	< 0.001
No	204 (73.1%)	51 (18.6%)		
Birth order				
First	29 (10.4%)	69 (24.9%)	59.47	< 0.001
Second	60 (21.5%)	79 (28.5%)		
Third	46 (16.5%)	70 (25.3%)		
Fourth and above	144 (51.6%)	59 (21.3%)		

### 3.3. Knowledge of Mothers on Complementary Feeding

In rural areas, 67.4% of respondents know the recommended age for continuing breastfeeding after 6 months, while 88.1% of urban respondents are aware. Regarding complementary food, 79.2% rural participants and 93.5% urban participants know when to start. For meal frequency, 77.1% of rural and 85.6% of urban respondents know the frequency for 6–8 months old, and 67% in rural vs. 81.3% in urban understand the frequency for 9–23 months old. Over half (58.1%) in rural and two‐thirds (70.4%) in urban areas recognize the importance of providing varied foods. Finally, 60.1% of rural mothers and 72.2% of urban mothers show sufficient knowledge regarding complementary feeding (Table 3).

**TABLE 3:** Mothers’ knowledge about complementary feeding.

Variables	Rural	Urban no (%)	*X* ^2^	*p* value
What should infants be fed just after birth?				
Breast milk	268 (96.1%)	277 (100)	11.42	0.001
Water	11 (3.9%)	0		
Until what age should a child enjoy exclusive breastfeeding				
< 6 months	24 (8.6%)	8 (2.9%)	24.09	< 0.001
At 6 months	221 (79.2%)	259 (93.5%)		
> 6 months	34 (12.2%)	10 (3.6%)		
Until what age is it recommended to continue breastfeeding after 6 months				
Up to one year	9 (3.2%)	13 (4.7%)	45.66	1.21
Up to 2 years	188 (67.4%)	244 (88.1%)		
Above 2 years	82 (29.4%)	20 (7.2%)		
Have you heard about complementary feeding practice?				
Yes	279 (100%)	277 (100%)	0	
No	0	0		
When should a child start complementary foods				
Before 6 months	24 (8.6%)	8 (2.9%)	24.09	< 0.001
At 6 months	221 (79.2%)	259 (93.5%)		
After 6 months	34 (12.2%)	10 (3.6%)		
How many times a day should a child be fed solid and/or semisolid food?				
Age 6–8 months				
One time	64 (22.9%)	40 (14.4%)	6.60	0.0101
≥ two times	215 (77.1%)	237 (85.6%)		
Age 9–23 months				
Two times	92 (33%)	52 (18.7%)	14.6	< 0.001
≥ three times	187 (67%)	225 (81.3%)		
Heard about the importance of providing different food groups to a child as complementary food				
Yes	162 (58.1%)	195 (70.4%)	9.19	0.002
No	117 (41.9%)	82 (29.6%)		
Do you know that bottle feeding may harm your child?				
Yes	57 (20.4%)	106 (38.3%)	21.34	< 0.001
No	222 (79.6%)	171 (61.7%)		
Mother’s knowledge about complementary feeding				
Sufficient knowledge	168 (60.1%)	200 (72.2%)	8.92	0.003
Insufficient knowledge	111 (39.9%)	77 (27.8%)		

### 3.4. Child Feeding Practices

All mothers in both areas have breastfed their children. Rural residents′ mothers (73.1%) and urban residents′ mothers (83.4%) initiated breastfeeding early within the first hour of delivery. Two‐thirds of rural (67.2%) and urban (73.8%) children began receiving complementary food at 6 months of age. About 72.4% of rural mothers used health extension workers for feeding information, while 37.5% of urban mothers relied on mass media. Among breastfed infants aged 6–8 months, 51.1% in rural and 76.6% in urban areas had two or more meals daily. Among children aged 9–23 months, 66.6% from rural and 83.3% from urban areas received three or more meals per day. Among non‐breastfed 6‐ to 23‐month‐old children, 35% in rural and 70.6% in urban areas had four or more meals daily. Nearly half (44.5%) of rural mothers and 89.5% of urban mothers included snacks between meals. Most mothers (71.5% rural, 74.5% urban) used cups with spoons to feed their children, while 4.7% of rural and 12.4% of urban respondents used bottle feeding to feed their children (Table 4).

**TABLE 4:** Child feeding practices of mothers.

Variables	Rural *n* (%)	Urban *n* (%)	*X* ^2^	*p* value
Ever breastfed				
Yes	279 (100.0%)	277 (100.0%)		
Practice of EBF				
Yes	192 (68.8%)	210 (75.8%)	3.39	0.065
No	87 (31.1%)	67 (24.2%)		
Start breastfeeding after delivery				
Immediately within 1 h	204 (73.1.0%)	231 (83.4%)	8.622	0.013
After first hour	59 (21.1%)	36 (13%)		
After one day	16 (5.7%)	10 (3.6%)		
Breastfeeding time per day				
Less than 4 times	8 (3.08%)	48 (18.5%)	154.290	< 0.001
4–6 times	30 (11.6%)	126 (48.5%)		
6–8 times	115 (44.4%)	61 (23.6%)		
More than 8 times	106 (40.9%)	25 (9.6%)		
Still breastfeeding your child				
Yes	259 (92.8%)	260 (93.9%)	0.238	0.626
No	20 (7.2%)	17 (6.1%)		
Why did you stop breastfeeding				
Lack of adequate breast milk	6 (30.0%)	3 (17.6%)	4.727	0.193
The child starts with solid and semisolid	3 (15.0%)	8 (47.1%)		
Due to pregnancy	10 (50.0%)	5 (29.4%)		
Work load	1 (5.0%)	1 (5.9%)		
Age of complementary feeding practice				
< 6 months	25 (9.1%)	53 (19.3%)	36.17	< 0.001
At 6 months	184 (67.2%)	203 (73.8%)		
> 6 months	65 (23.7%)	19 (6.9%)		
Who decides to give semisolid or soft foods				
Mother	232 (84.6%)	255 (92.8%)	12.18	0.006
Father	11 (4.01%)	10 (3.6%)		
Grand mother	12 (4.8%)	5 (1.8%)		
HEW/health professional	19 (6.9%)	5 (1.8%)		
Source of information on complementary feeding				
Health extension worker	202 (72.4%)	55 (19.9%)	184.08	< 0.001
Health professionals	26 (9.3%)	73 (26.4%)		
Mass media/radio/TV	10 (3.6)	104 (37.5%)		
Family	41 (14.7%)	45 (16.2%)		
What made you decide to start giving foods to your infant?				
Not enough breast milk	78 (28.6%)	40 (14.4%)	79.01	< 0.001
Because told to start	68 (24.7%)	17 (6.5%)		
Not enough time to breastfeed	11 (43%)	60 (22.02%)		
Baby reaching for food	117 (42.3%)	158 (57%)		
Complementary feeding frequency among breastfed children				
At 6–8 months of age (2–3 times per day)	22 (51.1%)	23 (76.6%)	0.89	0.64
Age 9–23 months (3‐4 times per day)	144 (66.6%)	190 (83.3%)		
Complementary feeding frequency among non‐breastfed children				
Age 6–23 months(≥ 4 times per day)	7 (35.0%)	12 (70.6%)		
Include snacks between meals				
Yes	122 (44.5%)	246 (89.5%)	125.79	< 0.001
No	152 (55.5%)	29 (10.5%)		
Type of complementary food started				
Gruel	96 (35.0%)	47 (17.09%)	64.46	5.55
Porridge	77 (28.1%)	114 (41.6%)		
Cow Milk	101 (36.6%)	73 (26.5%)		
Infant formula	0	41 (14.9%)		
Type of feeding containers used for the child				
Bottle	13 (4.7%)	34 (12.4%)	17.9	< 0.001
Cup	65 (23.7%)	36 (13.1%)		
Cup with spoon	196 (71.5%)	205 (74.5%)		
Do you wash hands before preparing food for your child				
Yes	271 (98.9%)	275 (100.0%)	3.02	0.081
No	3 (1.1%)	0		

### 3.5. Optimal Complementary Feeding Practice Indicators

Complementary feeding practice prevalence is significantly different among rural 13.1% and urban 25.1% children, respectively (*p* < 0.001).

The prevalence of children who initiated complementary feeding at the recommended time was 67.2% and 73.8% among rural and urban respondents, respectively. Among children, the proportion who received a minimum meal frequency was 63.1% among rural respondents and 72.5% among urban respondents. In terms of dietary diversity, the proportion of children who received minimum dietary diversity was 16.4% among rural respondents and 31.6% among urban respondents. Fifteen percent of rural respondents and 30.2% of urban respondents received the recommended minimum acceptable diet. Overall, children living in urban areas were 2.2 times more likely to practice appropriate complementary feeding than those in rural settings (AOR = 2.2, 95% CI: 1.42–3.452).

### 3.6. Consumed Food Groups Among Children in the 24 h Preceding Survey

During the 24 h preceding the survey, grains, roots, and tubers were the most commonly consumed food groups among both rural (83.2%) and urban (89.8%) children, which was followed by legumes and nuts (56.2% and 70.9%), dairy products (60.4% and 52.7%), vitamin A–rich foods (41.2% and 50.2%), egg (28.1% and 38.9%), other fruits and vegetables (20.4% and 25.1%) among rural and urban children, respectively. Meat and fish were the least commonly consumed food items, reported by only 2.6% of rural and 12.4% of urban children aged 6–23 months during the 24 h preceding, among the rural and urban respondents, respectively. Legumes, nuts, meats, and fish were consumed significantly in higher amounts by urban children than by rural children (*p* < 0.001) (Table 5).

**TABLE 5:** Type of food groups given to children.

Variables	Rural *N* (%)	Urban *N* (%)	*X* ^2^	*p* value
Grains, roots, and tubers				
Yes	228 (83.2%)	247 (89.8%)	5.137	0.023
No	46 (16.8%)	28 (10.2%)		
Legumes and nuts				
Yes	154 (56.2%)	195 (70.9%)	12.815	< 0.001
No	120 (43.8%)	80 (29.1%)		
Milk and milk products				
Yes	165 (60.4%)	145 (52.7%)	3.317	0.069
No	108 (39.6%)	130 (47.3%)		
Meat and fish				
Yes	7 (2.6%)	34 (12.4%)	19.109	< 0.001
No	267 (97.4%)	241 (87.6%)		
Eggs				
Yes	77 (28.1%)	107 (38.9%)	7.194	0.007
No	197 (71.9%)	168 (61.1)		
Vitamin A and vegetables				
Yes	113 (41.2%)	138 (50.2%)	4.421	0.035
No	161 (58.8%)	137 (49.8%)		
Other fruits and vegetables				
Yes	56 (20.4%)	69 (25.1%)	1.690	0.194
No	218 (79.6%)	206 (74.9%)		

### 3.7. Optimal Complementary Feeding Practice Determinants Among Children in Rural Areas

Binary logistic regression analysis revealed that optimal complementary feeding practices among rural respondents were associated with mother and father education, postnatal care, birth order, family size, maternal knowledge, and household security status. However, in the multivariable binary logistic regression model, only maternal knowledge and household security status were significantly associated with optimal complementary feeding practice in rural areas.

Mothers who knew the recommended complementary feeding practices were 3.7 times more likely to feed their children optimally than those who did not have sufficient knowledge (AOR = 3.7; 95% CI: 1.318–10.81; *p* < 0.05).

Food‐secured households with mothers were 3.5 times more likely to feed their children optimally than mothers from food insecure household (AOR = 3.5; 95% CI: 1.4–8.75; *p* < 0.001) (Table 6).

**TABLE 6:** Optimal complementary feeding practice determinants among respondents in rural area.

Variables	Optimal complementary feeding practice		COR (95% CI)	AOR (95% CI)	*p* value
	Yes (%)	No (%)			
Mother’s educational level					
Unable to read and write	6 (8.5%)	65 (91.5%)	1	1	
Read and write	5 (7.6%)	61 (92.4%)	0.888 (0.258–3.060)	1.06 (0.26–4.31)	0.927
Primary school	14 (14.4%)	83 (85.6%)	1.827 (0.66–5.017)	1.23 (0.39–3.86)	0.717
Secondary school	10 (27.8%)	26 (72.2%)	4.167 (1.374–12.63)	2.64 (0.71–9.78)	0.148
College/university	1 (25.0%)	3 (75.0%)	3.611 (0.323–40.32)	0	0.999
Father’s educational status					
Unable to read and write	3 (7.1%)	39 (92.9%)	1	1	
Read and write	5 (10.2%)	44 (89.8%)	1.477 (0.331–6.587)	0.92 (0.18–4.73)	0.920
Primary school	4 (4.7%)	81 (95.3%)	0.642 (0.137–3.009)	0.41 (0.07–2.28)	0.306
Secondary school	19 (23.2%)	63 (76.8%)	3.92 (1.08–14.122)	2.3 (0.55–9.65)	0.251
College/university	3 (42.9%)	4 (57.1%)	9.75 (1.455–65.35)	3.12 (0.34–8.78)	0.316
Postnatal care					
Yes	15 (22.7%)	51 (77.3%)	2.619 (1.260–5.442)	1.58 (0.60–4.17)	0.357
No	21 (10.1%)	187 (89.9%)	1	1	
Birth order					
First	7 (24.1%)	22 (75.9%)	3.702 (1.296–10.58)	4.92 (0.39–60.8)	0.215
Second	10 (16.7%)	50 (83.3%)	2.327 (0.931–5.820)	2.89 (0.49–17.1)	0.240
Third	8 (17.4%)	38 (82.6%)	2.450 (0.919–6.528)	1.75 (0.28–11.0)	0.550
Fourth and above	11 (7.9%)	128 (92.1%)	1	1	
Family size					
1–3	5 (25.0%)	15 (75.0%)	3.917 (1.130–13.57)	0.64 (0.03–13.5)	0.774
4–6	23 (15.1%)	129 (84.9%)	2.095 (0.898–4.888)	0.84 (0.15–4.67)	0.847
≥ 7	8 (7.8%)	94 (92.2%)	1	1	
Mothers’ knowledge					
Sufficient knowledge	29 (17.8%)	134 (82.2%)	3.75 (1.502–9.37)	3.7 (1.31–10.81)^∗^	0.013
Insufficient knowledge	6 (5.5%)	104 (94.5%)	1	1	
Household food security status					
Food secure	21 (25.0%)	63 (75.0%)	3.889 (1.888–8.009)	3.53 (1.4–8.75)^∗^	0.006
Food insecure	15 (7.9%)	175 (92.1%)	1	1	

Abbreviations: AOR, adjusted odds ratio; CI, confidence interval; COR, crude odds ratio.

^∗^ *p* value < 0.05.

### 3.8. Optimal Complementary Feeding Practice Determinants Among Children in Urban Areas

In the bivariate logistic regression analysis, maternal education, maternal occupation, postnatal care, child age, birth order, and maternal knowledge were significantly associated with optimal complementary feeding practices in urban areas, while in the multivariable binary logistic regression model, child age, postnatal care, and mothers’ knowledge revealed significant association with optimal complementary feeding practices in urban areas.

Mothers who had sufficient knowledge about optimal complementary feeding were 4.1 times more likely to feed their children optimally than mothers who did not have sufficient knowledge (AOR = 4.1; 95% CI: 1.74–9.62; *p* < 0.05).

Mothers who attended postnatal care were 2.58 times more likely to practice optimal complementary feeding than mothers who did not attend PNC (AOR = 2.58; 95% CI: 1.28–5.16; *p* < 0.01).

Mothers with children aged 12–23 months were 3.57 times more likely to practice optimal complementary feeding than their peers (AOR = 3.57; 95% CI: 1.08–11.805; *p* < 0.05) (Table 7).

**TABLE 7:** Determinants of optimal complementary feeding practice determinants in urban areas.

Variables	Optimal complementary feeding practice		COR (95% CI)	AOR (95% CI)	*p* value
	Yes (%)	No (%)			
Mother’s educational level					
Unable to read and write	1 (5.3%)	18 (94.7%)	1	1	
Read and write	3 (8.8%)	31 (91.2%)	1.74 (0.168–18.020)	1.01 (0.089–11.51)	0.993
Primary school	16 (27.6%)	42 (72.4%)	6.86 (0.844–55.683)	5.94 (0.661–53.34)	0.112
Secondary school	24 (27.0%)	65 (73%)	6.65 (0.841–52.531)	4.19 (0.476–36.89)	0.197
College/university	25 (33.3%)	50 (66.7%)	9.00 (1.136–71.331)	4.38 (0.441–43.63)	0.207
Mother occupation					
House wife	21 (17.9%)	96 (82.1%)	1	1	
Day laborer	1 (5.6%)	17 (94.4%)	0.27 (0.034–2.134)	0.15 (0.018–1.391)	0.096
Merchant	19 (32.2%)	40 (67.8%)	2.17 (1.055–4.470)	1.86 (0.798–4.353)	0.150
Government worker	28 (34.6%)	53 (65.4%)	2.41 (1.251–4.662)	1.91 (0.660–5.534)	0.233
Postnatal care					
Yes	52 (30.4%)	119 (69.6%)	2.47 (1.324–4.608)	2.57 (1.283‐5.159)^∗^	0.008
No	17 (16.3%)	87 (83.7%)	1	1	
Child age					
6–8 months	4 (13.3%)	26 (86.7%)	1	1	
9–11 months	14 (15.4%)	77 (84.6%)	1.182 (0.357–3.911)	1.78 (0.477–6.650)	0.39
12–23 months	51 (33.1%)	103 (66.9%)	3.218 (1.066–9.716)	3.57 (1.081‐11.81)^∗^	0.037
Birth order					
First	19 (27.9%)	49 (72.1%)	1.059 (0.516–2.173)	1.50 (0.652–3.459)	0.406
Second	23 (29.1%)	56 (70.9%)	0.287 (0.112–0.736)	0.36 (0.126–1.015)	0.954
Third	7 (10.0%)	63 (90.0%)	1.357 (0.636–2.895)	1.46 (0.595–3.608)	0.013
Fourth and above	20 (34.5%)	38 (65.5%)	1	1	
Mothers’ knowledge					
Sufficient knowledge	61 (30.7%)	138 (69.3%)	3.75 (1.702–8.296)	4.09 (1.74‐9.62)^∗^	< 0.001
Insufficient knowledge	8 (10.5%)	68 (89.5%)	1	1	

Abbreviations: AOR, adjusted odds ratio; CI, confidence interval; COR, crude odds ratio.

^∗^ *p* value < 0.05.

## 4. Discussion

Over the past 20 years, the WHO has developed a number of strategies to achieve optimal complementary feeding practices (≥ 80%) [8]. The current study compared optimal complementary feeding practices’ magnitude and its determinants among mothers of children aged 6–23 months in urban and rural settings of Durame and Kedida Gamela, revealing rates of 25.1% and 13.1%, respectively, with an overall prevalence of 19.1%. A significant difference was noted (*p* < 0.001), with higher optimal practices in urban areas. This may be attributed to better food security, socioeconomic status, exposure to feeding practice information, and healthcare facilities.

Compared to the study carried out in the South Wollo Zone, near Dessie Town Z in Ethiopia, the study’s pool prevalence of complementary feeding practices is higher (18.1%) among mothers with children aged 6 to 23 months [26]. The disparity could be explained by the time and location differences.

The study reveals that the optimal complementary feeding practice in rural areas is 13.1%, higher than findings in Damot Sore (11.4%) [27] and Southern Ethiopia (9.5%) [28]. However, the figure is lower than that in rural Sidama (14.4%) [29], Northern Ghana (15.7%) [30], and Bangladesh (20%) [31]. Similarly, the prevalence observed in urban areas (25.1%) was higher than that reported in Assela town (23.4%) [32] and Abyi Adi town (19.7%) [33], but lower than the findings from Ambo town (26.8%) [22], Dejen District (52%) [34], and Debre Berhan town (70.3%) [35]. This could be attributed to differences in the sample size, period of study, and sociocultural and socioeconomic status of the respondents.

In both rural (67.2%) and urban (73.3%) areas, more than half of participants in the present study began optimal feeding practice for their children at the age of 6 months. The magnitude of timely initiation of complementary feeding in the rural and urban areas was comparable to findings from studies conducted in Northwest Ethiopia (67.5%) [35] and the Damot Sore District (74.2%) [26], respectively. However, these findings were lower than those reported in studies conducted in Abyi Adi town (79.7%) [33], Ambo town (81.1%) [22], Sri Lanka (75.5%) [31], and Madhya Pradesh, India (74.2% rural area vs. 92.5% in urban area) [36], but higher than findings from a study conducted in Karnataka district of India (28.8% in urban area vs 42.11% in rural area) [37]. The reason for the difference noticed may be due to the difference in the culture of society; i.e., most commonly, they had done so as per the elder’s advice.

According to the current study, the percentages of minimum meal frequency were 72.5% in urban areas and 63.1% in rural areas. In comparison with the nationwide prevalence reported in the EDHS 2016 for both rural and urban areas (43.2% rural vs. 59.3% urban), higher figures were found in this study [18]. The possible reason for this disparity might be due to the fact that the EDHS were conducted among a culturally different population in different regions of the country with a wide variety of child feeding practices. Similarly, the current findings were higher compared to study conducted from Nigeria (33.1% rural vs 51.2% urban) [38], India (41.5% in rural and 71.5% in urban) [39], Bensa district (53.2%) [40], and lower than the report from Madhya Pradesh, India (88.1% in rural area, 90.1% in Urban area) [36], Ambo town (81.5%) [22], Harar (76%) [41], rural Damot Sore (94.5%) [27], and Bangladesh (81%) [31]. The possible reason for the differences could be due to variability in livelihoods and differences in the dissemination of information on child feeding practices and socioeconomic differences.

There was a significant difference (*p* < 0.001) between the minimum meal frequency in rural areas (63.1%) and urban areas (72.5%). This indicates that urban children are fed more frequently, likely due to better food availability and economic status. Furthermore, the minimum dietary diversity was met by 31.6% of urban children and 16.5% of rural children. Urban figure aligns with the EDHS 2016 report for urban Ethiopia (30.5%), while the rural percentage exceeds the rural figure in the same report (11.5%) [18]. This study is higher than that reported in a study conducted in Bensa district (20.4%) [40], rural Gorche district (10.6%) [42], Abyi Adi town (10.8%) [33], and lower than finding from Karnataka, India (19.47% in rural and 54.75% in urban) [39], and East Delhi (32.6%) [43]. Minimum dietary diversity in rural areas (16.4%) was significantly lower than the urban areas (31.6%) (*p* < 0.001). The possible reason could be due to diversified food availability at home, family’s income, and mothers who are living in urban areas have better awareness of diversified dietary practice compared to rural mothers.

The proportion of mothers who practiced provision of a minimum acceptable diet to their children in rural and urban areas, respectively, was 15% and 30.2% in this study. This figure was higher than that of the report from national prevalence reported in the EDHS 2016 for rural and urban areas (5.7% and 18.5%, respectively) [18], rural Goncha district (12.6%) [44], Bensa district (16%) [40], rural Damot Sore (16.3%) [27], Abyi Adi town (11.9%) [33], Nepal (26.5%) [45], and lower than study finding from Karnataka, India (15.8% in rural and 40% in urban) [39], and Sri Lanka (68%) [31]. The difference could be due to cultural background and differences in educational levels.

Mothers who attended postnatal care services in urban areas were 2.6 times more likely to practice optimal complementary feeding compared to those who did not receive postnatal care. This finding is consistent with research from Abyi Adi town [33], Enemay district [46], and Lasta district [47], which shows a significant correlation between inadequate complementary feeding practices and the lack of postnatal follow‐up. This could be because of healthcare providers’ education and counseling services on child feeding practices during follow‐up, and it helps to avoid cultural beliefs that hinder child feeding practices. Lack of postnatal follow‐up was identified as the primary risk factor for inappropriate feeding practices according to the study in Tanzania [48]. To enhance mothers’ IYCF knowledge and appropriate complementary feeding practice, they should be encouraged to attend antenatal and postnatal services.

Compared to infants in the 6‐ to 11‐month age group, children in the 18‐ to 23‐month age group are 3.6 times more likely to be fed optimally. The results from northwest Ethiopia [49], southern Ethiopia [28], and Sri Lanka [31] are comparable to this one. This might be attributed to mothers’ misconceptions that when a younger baby begins receiving complementary foods, they only include formula or cow’s milk. Another possibility is that mothers believe younger infants’ stomachs are unable to digest solid foods. This implies that mothers’ knowledge of newborn and child feeding practices should be prioritized by health planners and implementers.

According to the study, mothers in urban areas with adequate knowledge are four times more likely to feed children optimally than those with inadequate knowledge (*p* < 0.001). In rural areas, a similar finding was observed that mothers with sufficient knowledge are 3.7 times more likely to feed children optimally than those with insufficient knowledge (*p* < 0.05). Other studies in Debre Birhan town [35], Jimma Arjo [50], and East Delhi [43] support these findings. This may be attributed to the efforts of health extension workers’ nutrition promotions that have enhanced maternal knowledge and positively impacted the feeding practices. Over half of mothers demonstrated sufficient knowledge both in rural (60.1%) and urban (72.2%) areas, though poor complementary food practices persisted in both settings.

The study found that food secure households in rural areas are 3.5 times more likely to practice optimal complementary feeding compared to food insecure households. It indicated that inadequate food availability negatively impacts feeding practices, while food secure households support timely initiation, dietary diversity, and meal frequency. Studies in the Bensa district [40] and Argentina [51] reinforce these findings.

### 4.1. Limitations of the Study

• The cross‐sectional study design used in this study does not allow for the establishment of a causal relationship.
• The 24‐h recall method may not accurately reflect participants’ usual past feeding and dietary habits as the study may be subject to recall and social desirability bias, which could affect the accuracy of the reported information.
• The study was done in only two districts; as a result, the generalizability of the findings to a wider population may be limited.

## 5. Conclusion

Optimal complementary feeding practice magnitude is significantly higher in urban areas compared to rural areas; however, the overall level of practice remained low in both settings.

Child age, postnatal care, and maternal knowledge are significantly associated with optimal complementary feeding practices in urban areas, while mothers’ knowledge and household food security status are the key associated factors in rural areas.

Mothers who utilized postnatal care services in urban areas are 2.6 times more likely to practice optimal complementary feeding compared to those who did not attend postnatal care.

## 6. Recommendation

Therefore, in light of the findings from this research, for healthcare workers,

• Enhance postnatal care services to strengthen and promote postnatal care utilization, especially in urban areas, as it significantly increases the likelihood of optimal complementary feeding in Durame Town and Kedida Gamela District, Southern Ethiopia.
• Improve maternal nutritional knowledge: Develop targeted education programs to increase maternal awareness of the importance of complementary feeding, focusing both in urban and rural areas of Durame Town and Kedida Gamela District.
• Integrating newborn and young child‐feeding programs with basic maternal health services is important.

For governmental and nongovernmental organizations,

• Focus on addressing the household food insecurity by enhancing the production and supply of food in the community via sectoral collaboration.
• Provide nutritional education/counseling for mothers to improve infant and young child feeding practices, particularly regarding the timing, variety, quantity, and frequency of complementary feeding.

For researchers,

• To identify barriers to optimal complementary feeding practice in a comprehensive way, a triangulated prospective study design should be used.

**Nomenclature**
ANC: Antenatal care
AOR: Adjusted odds ratio
CF: Complementary feeding
CI: Confidence interval
COR: Crude odds ratio
CSA: Central Statistical Agency
DDS: Dietary diversity score
EBF: Exclusive breastfeeding
EDHS: Ethiopian Demographic and Health Survey
FADUA: Frequency, adequacy, density, utilization, and active feeding
FANTA: Food and Nutrition Technical Assistance
FAO: Food and Agriculture Organization
FDRE: Federal Democratic Republic of Ethiopia
FMOH: Federal Ministry of Health
HFIAS: Household Food Insecurity Access Scale
IYCFP: Infant and Young Child Feeding Practice
PNC: Postnatal care
SPSS: Statistical Package for Social Science
SSA: Sub‐Saharan Africa
UNICEF: United Nations Children’s Fund
USAID: United States Agency for International Development
WHO: World Health Organization

## Funding

No funding was received for this manuscript.

## Ethics Statement

Ethical clearance was given by the Institutional Review Board (IRB) of Hawassa University Medicine and Health Science College. A letter asking for authorization was sent to the Durame town and Kedida Gamela District Health offices. Then, permission and support letters were obtained from two district administration offices, including the kebeles, the smallest administrative unit, from which household surveys were conducted. Participants were given a detailed explanation about the purpose of the study. The respondents provided their informed oral consent before data collection. Confidentiality of all information was ensured. Respondents’ freedom to decline participation at any stage of the interview process was upheld.

## Conflicts of Interest

The authors declare no conflicts of interest.

## Data Availability

The original data from this study may be available to third parties upon request to the authors.

## References

[1] WHO , Indicators for Assessing Infant and Young Child Feeding Practices: Part 1: Definitions: Conclusions of a Consensus Meeting Held 6-8 November 2007 in Washington DC, USA, 2008.

[2] Black R. E. , Allen L. H. , Bhutta Z. A. et al., Maternal and Child Undernutrition: Global and Regional Exposures and Health Consequences, The Lancet. (2008) 371, no. 9608, 243–260, 10.1016/s0140-6736(07)61690-0, 2-s2.0-38349061342. 18207566

[3] Children S. T. , State of the World’s Mothers 2012: Nutrition in the First 1,000 Days, 2012.

[4] WHO, Essential Nutrition Actions: Improving Maternal, Newborn, Infant and Young Child Health and Nutrition, 2013. 25473713

[5] British Dietetic Association , Complementary Feeding: Introduction of Solid Food to an Infants Diet, Policy statement Birmingham. United Kingdom: The British Dietetic Association. (2013) .

[6] Hawkes C. , Demaio A. R. , and Branca F. , Double-Duty Actions for Ending Malnutrition within a Decade, Lancet Global Health. (2017) 5, no. 8, e745–e746, 10.1016/s2214-109x(17)30204-8, 2-s2.0-85019833133. 28528865

[7] Unicef %J Unived and New York N. , USA, Levels and Trends in Child Malnutrition: Joint Child Malnutrition Estimates, 2021.

[8] Who , Global Strategy for Infant and Young Child Feeding, 2003, World Health Organization.

[9] Black R. et al., Executive Summary of the Lancet Maternal and Child Nutrition Series, Maternal and Child Nutrition. (2013) 1–12.

[10] Harttgen K. , Klasen S. , and Vollmer S. , Economic Growth and Child Undernutrition in Sub‐Saharan Africa, Population and Development Review. (2013) 39, no. 3, 397–412, 10.1111/j.1728-4457.2013.00609.x, 2-s2.0-84884128445.

[11] Islam M. A. , Assessment of Nutritional Status and Prevalence of Wasting, Stunting and Underweight of Forcibly Displaced Rohingya Children 6-59 Months of Age Living Makeshift Camp, Cox’s Bazar, Bangladesh: a Cross-Sectional Study, 2022.

[12] Tedla M. , Malede A. , and Berhan Z. J. T. P. A. M. J. , Prevalence and Associated Factors of Malnutrition Among Under-five Children Living in Slum Areas of Bahir Dar Town, The Pan African Medical Journal. (2024) 47, Ethiopia, 10.11604/pamj.2024.47.176.33439. PMC11260059 39036031

[13] Unicef , Levels & Trends in Child Mortality: Report 2015: Estimates/Developed by the UN Inter-Agency Group for Child Mortality Estimation, 2015.

[14] Onyango A. W. , Borghi E. , de Onis M. , Casanovas MdC. , and Garza C. , Complementary Feeding and Attained Linear Growth Among 6–23-month-old Children, Public Health Nutrition. (2014) 17, no. 9, 1975–1983, 10.1017/s1368980013002401, 2-s2.0-84905233245. 24050753 PMC11108726

[15] Lartey A. , Maternal and Child Nutrition in Sub-Saharan Africa: Challenges and Interventions, Proceedings of the Nutrition Society. (2008) 67, no. 1, 105–108, 10.1017/s0029665108006083, 2-s2.0-38749115865. 18234138

[16] Assefa Y. et al., Successes and Challenges of the Millennium Development Goals in Ethiopia: Lessons for the Sustainable Development Goals, 2017. 10.1136/bmjgh-2017-000318 PMC5656143 29081999

[17] Tololu A. K. , Teshome B. , Fessaha H. Z. , and Kaso A. W. , Determinants of Appropriate Complementary Feeding Practices Among Mothers of Children Aged 6–23 Months in Bokoji Town, Oromia Region, Ethiopia, Determinants of appropriate complementary feeding practices among mothers of children aged 6–23 months in Bokoji town, Oromia region, Ethiopia. (2025) 25, no. 1, 10.1186/s12887-025-05443-9. PMC11783710 39891126

[18] Csa , Ethiopia Demographic and Health Survey 2016, Addis Ababa, Ethiopia, and Rockville, Maryland, USA, 2017, CSA and ICF.

[19] Fdre , National Nutrition Programme II 2016-2020-1.pdf>, 2016.

[20] Ayele S. , Zegeye E. A. , and Nisbett N. J. B. I. , Multi-Sectoral Nutrition Policy and Programme Design, Coordination and Implementation in Ethiopia, 2020.

[21] Areja A. , Yohannes D. , and Yohannis M. J. B. n. , Determinants of Appropriate Complementary Feeding Practice Among Mothers Having Children 6–23 Months of Age in Rural Damot Sore District, Southern Ethiopia; a Community Based Cross Sectional Study, 2017. 10.1186/s40795-017-0202-y PMC7050925 32153858

[22] Terfassa T. G. , Complementary Feeding Practices and Associated Factors Among Mothers Having Children 6-23 Months of Age in Ambo Town, West Shoa Zone, Oromia, Ethiopia, EC Nutrition. (2020) 15, 01–11.

[23] Coates J. , Swindale A. , and Bilinsky P. , Household Food Insecurity Access Scale (HFIAS) for Measurement of Food Access: Indicator Guide, 2007.

[24] Organization W. H. , Indicators for Assessing Infant and Young Child Feeding Practices: Part 2: Measurement, 2010, World Health Organization.

[25] WHO Guideline for Complementary Feeding of Infants and Young Children 6–23 Months of Age, 2023.

[26] Belete S. , Kebede N. , Chane T. , Melese W. , and Tadesse S. E. , Optimal Complementary Feeding Practices and Associated Factors Among Mothers Having Children 6 to 23 Months, South WOLLO Zone, Dessie ZURIA, Ethiopia, 2022, 10.1016/j.pedn.2022.08.021. 36115754

[27] Areja A. , Yohannes D. , and Yohannis M. , Determinants of Appropriate Complementary Feeding Practice Among Mothers Having Children 6–23 Months of Age in Rural Damot Sore District, Southern Ethiopia; a Community Based Cross Sectional Study, BMC Nutrition. (2017) 3, no. 1, 10.1186/s40795-017-0202-y. PMC7050925 32153858

[28] Kassa T. , Meshesha B. , Haji Y. , and Ebrahim J. , Appropriate Complementary Feeding Practices and Associated Factors Among Mothers of Children Age 6–23 Months in Southern Ethiopia, 2015, BMC Pediatrics. (2016) 16, no. 1, 10.1186/s12887-016-0675-x, 2-s2.0-84982275898. PMC4992197 27542833

[29] Tessema M. , Belachew T. , and Ersino G. , Feeding Patterns and Stunting During Early Childhood in Rural Communities of Sidama, South Ethiopia, Pan African Medical Journal. (2013) 14, no. 1, 10.11604/pamj.2013.14.75.1630, 2-s2.0-84876481332. PMC3641921 23646211

[30] Saaka M. , Wemakor A. , Abizari A. R. , and Aryee P. , How Well do WHO Complementary Feeding Indicators Relate to Nutritional Status of Children Aged 6–23 Months in Rural Northern Ghana?, BMC Public Health. (2015) 15, no. 1, 1–12, 10.1186/s12889-015-2494-7, 2-s2.0-84959119982. 26596246 PMC4656186

[31] Senarath U. , Agho K. E. , Akram D. et al., Comparisons of Complementary Feeding Indicators and Associated Factors in Children Aged 6–23 Months Across Five South Asian Countries, Maternal and Child Nutrition. (2012) 8, no. s1, 89–106, 10.1111/j.1740-8709.2011.00370.x, 2-s2.0-83755185649. 22168521 PMC6860856

[32] Sasie S. , Oljira L. , and Demena M. , Infant and Young Child Feeding Practice and Associated Factors Among mothers/caretakers of Children Aged 0–23 Months in Asella Town, South East Ethiopia, Journal of Family Medicine. (2017) 4, no. 5, 11–22.

[33] Mekbib E. et al., Magnitude and Factors Associated with Appropriate Complementary Feeding Among Mothers Having Children 6–23 months-of-age in Northern Ethiopia; a Community-based cross-sectional Study, Journal of Food and Nutrition Sciences. (2014) 2, no. 2, 10.11648/j.jfns.20140202.13.

[34] Ww B. and Kk A. , Complementary Feeding Practice and Associated Factors Among Mothers of Children 6-23 Months of Age in Dejene District, Northwest, Ethiopia 2015, 2017, Journal of community medicine & health education, 1–4.

[35] Asfaw T. , Prevalence and Determinants of Appropriate Child Feeding Practice Among Mothers Having Children with and Without Diarrhea Aged 6–23 Months in Debre Berhan Town, Ethiopia, Research and Reports in Neonatology. (2021) 11, 35–42, 10.2147/rrn.s289640.

[36] Yadav Ys Y. S. , Rathi S. , Dhaneria M. , Singh P. , and Singh D. P. , Comparison of Infant Feeding Practices Among Rural and Urban Mothers: an Observational Study, Int Journal of Medical Research. (2015) 3, no. 6, 547–553, 10.17511/ijmrr.2015.i6.104.

[37] Ashwini S. , Katti S. , and Mallapur M. J. A. A. J. M. S. , Comparison of Complementary Feeding Practices Among Urban and Rural Mothers–a Cross Sectional Study, 2014.

[38] Olumodeji O. O. , A Comparative Study of Complementary Feeding and Nutritional Status of Children Aged 6-24 Months in A Rural and an Urban Lga of Lagos State, Public Health. (2015) .

[39] Katti S. , Mallapur M. , and Ashwini S. , Comparison of Complementary Feeding Practices Among Urban and Rural mothers-a Cross Sectional Study, 2014.

[40] Birhanu M. , Abegaz T. , and Fikre R. , Magnitude and Factors Associated with Optimal Complementary Feeding Practices Among Children Aged 6-23 Months in Bensa District, Sidama zone, South Ethiopia, Ethiopian Journal of Health Sciences. (2019) 29, no. 2, 10.4314/ejhs.v29i2.2, 2-s2.0-85065115386. PMC6460456 31011263

[41] Semahegn A. , Tesfaye G. , and Bogale A. , Complementary Feeding Practice of Mothers and Associated Factors in Hiwot Fana Specialized Hospital, Eastern Ethiopia, The Pan African Medical Journal. (2014) 18, 10.11604/pamj.2014.18.143.3496, 2-s2.0-84902985717. PMC4236776 25419281

[42] Dangura D. and Gebremedhin S. , Dietary Diversity and Associated Factors Among Children 6-23 Months of Age in Gorche District, Southern Ethiopia: Cross-Sectional Study, BMC Pediatrics. (2017) 17, no. 1, 1–7, 10.1186/s12887-016-0764-x, 2-s2.0-85011396196. 28068965 PMC5223415

[43] Khan A. M. , Kayina P. , Agrawal P. , Gupta A. , and Kannan A. , A Study on Infant and Young Child Feeding Practices Among Mothers Attending an Urban Health Center in East Delhi, Indian Journal of Public Health. (2012) 56, no. 4, 10.4103/0019-557x.106420, 2-s2.0-84875891668. 23354143

[44] Birie B. , Kassa A. , Kebede E. , and Terefe B. , Minimum Acceptable Diet Practice and Its Associated Factors Among Children Aged 6–23 Months in Rural Communities of Goncha District, North West Ethiopia, BMC Nutrition. (2021) 7, no. 1, 10.1186/s40795-021-00444-0. PMC8290531 34281613

[45] Khanal V. , Sauer K. , and Zhao Y. , Determinants of Complementary Feeding Practices Among Nepalese Children Aged 6–23 Months: Findings from Demographic and Health Survey 2011, BMC Pediatrics. (2013) 13, no. 1, 10.1186/1471-2431-13-131, 2-s2.0-84883067061. PMC3766108 23981670

[46] Gessese D. et al., The Practice of Complementary Feeding and Associated Factors Among Mothers of Children 6-23 Months of Age in Enemay District, Northwest Ethiopia, Nutrition & Food Science. (2014) .

[47] Molla M. , Ejigu T. , and Nega G. , Complementary Feeding Practice and Associated Factors Among Mothers Having Children 6–23 Months of Age, Lasta District, Amhara Region, Northeast Ethiopia, Advances in Public Health. (2017) 2017, 1–8, 10.1155/2017/4567829.

[48] Mwita L. O. , Correlates of Complementary Feeding Practice Among Caregivers of Infants and Young Children Aged 6-24 Months at Mbagathi District Hospital, Nairobi, 2014, University of Nairobi.

[49] Beyene M. , Worku A. G. , and Wassie M. M. , Dietary Diversity, Meal Frequency and Associated Factors Among Infant and Young Children in Northwest Ethiopia: a cross-sectional Study, BMC Public Health. (2015) 15, no. 1, 10.1186/s12889-015-2333-x, 2-s2.0-84943454327. PMC4592571 26433689

[50] Tamiru D. , Aragu D. , and Belachew T. , Survey on the Introduction of Complementary Foods to Infants Within the First Six Months and Associated Factors in Rural Communities of Jimma Arjo, International Journal of Nutrition and Food Sciences. (2013) 2, no. 2, 77–84.

[51] Lindsay A. C. , Ferarro M. , Franchello A. et al., Child Feeding Practices and Household Food Insecurity Among low-income Mothers in Buenos Aires, Argentina, Ciência & Saúde Coletiva. (2012) 17, no. 3, 661–669, 10.1590/s1413-81232012000300012, 2-s2.0-84859037551. 22450407

